# Inhibition of Matrix Metalloproteinase 9 Activity Promotes Synaptogenesis in the Hippocampus

**DOI:** 10.1093/cercor/bhab050

**Published:** 2021-03-19

**Authors:** Ahmad Salamian, Diana Legutko, Klaudia Nowicka, Bogna Badyra, Paulina Kaźmierska-Grębowska, Bartosz Caban, Tomasz Kowalczyk, Leszek Kaczmarek, Anna Beroun

**Affiliations:** Laboratory of Neurobiology, Nencki-EMBL Center of Excellence for Neural Plasticity and Brain Disorders: BRAINCITY, Nencki Institute of Experimental Biology of the Polish Academy of Sciences, Warsaw 02-093, Poland; Laboratory of Molecular Basis of Behavior, Nencki Institute of Experimental Biology of the Polish Academy of Sciences, Warsaw 02-093, Poland; Laboratory of Neurobiology, Nencki-EMBL Center of Excellence for Neural Plasticity and Brain Disorders: BRAINCITY, Nencki Institute of Experimental Biology of the Polish Academy of Sciences, Warsaw 02-093, Poland; Laboratory of Neurobiology, Nencki-EMBL Center of Excellence for Neural Plasticity and Brain Disorders: BRAINCITY, Nencki Institute of Experimental Biology of the Polish Academy of Sciences, Warsaw 02-093, Poland; Laboratory of Neurobiology, Nencki-EMBL Center of Excellence for Neural Plasticity and Brain Disorders: BRAINCITY, Nencki Institute of Experimental Biology of the Polish Academy of Sciences, Warsaw 02-093, Poland; Department of Neurobiology, Faculty of Biology and Environmental Protection, University of Lodz, Lodz 90-236, Poland; Department of Neurobiology, Faculty of Biology and Environmental Protection, University of Lodz, Lodz 90-236, Poland; Department of Neurobiology, Faculty of Biology and Environmental Protection, University of Lodz, Lodz 90-236, Poland; Laboratory of Neurobiology, Nencki-EMBL Center of Excellence for Neural Plasticity and Brain Disorders: BRAINCITY, Nencki Institute of Experimental Biology of the Polish Academy of Sciences, Warsaw 02-093, Poland; Laboratory of Neurobiology, Nencki-EMBL Center of Excellence for Neural Plasticity and Brain Disorders: BRAINCITY, Nencki Institute of Experimental Biology of the Polish Academy of Sciences, Warsaw 02-093, Poland; Laboratory of Neuronal Plasticity, Nencki-EMBL Center of Excellence for Neural Plasticity and Brain Disorders: BRAINCITY, Nencki Institute of Experimental Biology of the Polish Academy of Sciences, Warsaw 02-093, Poland

**Keywords:** carbachol, excitatory, inhibitory, MMP-9, synaptogenesis

## Abstract

Information coding in the hippocampus relies on the interplay between various neuronal ensembles. We discovered that the application of a cholinergic agonist, carbachol (Cch), which triggers oscillatory activity in the gamma range, induces the activity of matrix metalloproteinase 9 (MMP-9)—an enzyme necessary for the maintenance of synaptic plasticity. Using electrophysiological recordings in hippocampal organotypic slices, we show that Cch potentiates the frequency of miniature inhibitory and excitatory postsynaptic currents (mIPSCs and mEPSCs, respectively) in CA1 neurons and this effect is MMP-9 dependent. Interestingly, though MMP-9 inhibition prevents the potentiation of inhibitory events, it further boosts the frequency of excitatory mEPSCs. Such enhancement of the frequency of excitatory events is a result of increased synaptogenesis onto CA1 neurons. Thus, the function of MMP-9 in cholinergically induced plasticity in the hippocampus is to maintain the fine-tuned balance between the excitatory and the inhibitory synaptic transmission.

## Introduction

Complex cognitive functions require orchestrated activity of various neuronal ensembles, distributed across the brain (Bland and Colom 1993; Buzsáki 2006). In the hippocampus, which is responsible for memory integration processes, such as encoding, short-term and long-term consolidation, and retrieval (Riedel and Micheau 2001; Huang and Kandel 2005), precise timing of neuronal discharges is essential to induce synaptic plasticity that modifies synaptic strength and allows information coding (O'Keefe and Recce 1993).

Hippocampal excitatory transmission is tightly controlled by the inhibitory as well as neuromodulatory systems, and cholinergic activation impacts on the threshold and the magnitude of plasticity at glutamatergic synapses (Drever et al. 2011). It has been shown that application of carbachol (Cch) induces LTP and increases dendritic spines turnover in the CA1 region (Huerta and Lisman 1993; Shinoe et al. 2005; De Roo et al. 2008). Moreover, several lines of evidence indicate that cholinergic activation, for example, via Cch, can modify excitatory as well as inhibitory synaptic transmission (Heister et al. 2009; Moriguchi et al. 2009; Kozhemyakin et al. 2010; Sun and Kapur 2012). Yet, the long-term consequences of such modulation have not been properly addressed. Therefore, in this study, we investigate how the cholinergically induced plasticity in slice preparations impact on inhibitory and excitatory networks within the hippocampus.

The extracellular matrix (ECM) creates an environment that integrates connectivity and activity of neural networks (Dityatev and Schachner 2003). Modification of ECM, which allows synaptic plasticity, orchestrates the function of complex networks of various types of neurons and glia (Song and Dityatev 2018). Thus, changes in ECM structure have a strong impact on hippocampal and cortical activity (Gurevicius et al. 2004; Gurevicius et al. 2009; Morellini et al. 2017). Matrix metalloproteinases are enzymes that modify ECM in a tightly controlled manner (Rivera et al. 2010; Huntley 2012). Of particular importance is an activity-dependent matrix metalloproteinase 9 (MMP-9) that in the hippocampus is required for the maintenance of LTP and dendritic spine remodeling (Nagy et al. 2006; Wang et al. 2008; Wiera et al. 2013; Szepesi et al. 2014; Magnowska et al. 2016; Vafadari et al. 2016) and has a strong impact on memory formation in the hippocampus-dependent tasks, such as Morris water maze and contextual fear conditioning (Meighan et al. 2006; Nagy et al. 2006; Brown et al. 2007; Wright et al. 2007).

Thus, we hypothesize that Cch-induced plasticity in hippocampal slices might affect MMP-9 activity and enhance dendritic spine turnover. We, therefore, have tested the requirement of MMP-9 function in Cch-induced plasticity in hippocampal organotypic slices and have discovered that though Cch stimulation induces MMP-9 activity, it is MMP-9 inhibition that profoundly increases the excitatory synaptic transmission in the hippocampus. This increase is driven by the enhanced formation of new glutamatergic synapses on CA1 pyramidal neurons.

## Materials and Methods

### Animals

Wistar rats used in this study were purchased from Mossakowski Medical Research Centre, Warsaw, Poland. MMP-9 knockout (MMP-9 KO) mice were housed at the Animal Facility of the Nencki Institute, Warsaw, Poland. The colony was maintained by breeding heterozygous mice. Their homozygous offspring (MMP-9 KOs and their WT siblings) were used in this study. The KO mice were genotyped using primers (forward: 5′ GAA GGG ACT GGC TGC TAT TG and reverse: 5′ AAT ATC ACG GGT AGC CAA CG) detecting the neomycin cassette, as this strain was generated by replacing a fragment of exon 2 and the entire intron 2 with a neomycin phosphotransferase cDNA-containing cassette (Vu et al. 1998). GAD65-tdTomato mice (Besser et al. 2015) were housed at the Animal Facility of the Nencki Institute. They were bred by crossing heterozygous mice with wild-type ones. Heterozygous offspring used in this study was genotyped with primers recognizing the tdTomato insert (forward: 5′ GTGCAGGGTCGAGGCAAAGGCA and reverse: 5′ GGACAGGATGTCCCAGGCGAAG).

### Organotypic Slice Culture

Transverse organotypic slice cultures (300–350 μm thickness) were prepared from postnatal day (P) 6–8 rats or P4–5 mice (Gogolla et al. 2006a, 2006b), according to the protocol approved by the Local Ethics Committee in Warsaw, Poland (no 574/2014). Animals were anesthetized with isoflurane and decapitated. After removing the brain and separating the two hemispheres, whole, intact hippocampi were dissected out and sliced sagittally with the McIlwain tissue chopper. Slices were separated from each other in the dissecting medium (49.4 mL Gey’s Balanced Salt Solution, 100 μL d-Glucose solution 45%, 500 μL HEPES solution 1 M). Next, slices with intact hippocampal structure collected in culture medium (23.7 mL Minimum Essential Medium Eagle, 12.5 mL Hank’s Balanced Salts Solution, 12.5 mL Horse serum, 100 μL d-Glucose solution 45%, 500 μL Penicillin-Streptomycin, 250 μL l-Glutamine 200 mM). Slices were transferred onto the interface membrane (cell culture insert, Millipore; PICM03050), which had been incubated (35 °C and 5% CO_2_) and supplied with 1 mL of culture medium in the 6-well culture plate. The cultures were placed in a CO_2_ incubator for 2 weeks, and the medium was exchanged twice a week.

### Electrophysiology

#### Whole-Cell Patch-Clamp Recording

The standard whole-cell patch-clamp recordings were performed with an ELC-03XS amplifier (npi). Data were acquired with custom algorithms in Igor Pro 6.2 (WaveMetrics). In order to perform the whole-cell recording, a piece of interface membrane containing a hippocampal organotypic slice was cut and transferred to a submerged recording chamber where it was continuously perfused with oxygenated (95% O_2_/5% CO_2_) ACSF (119 mM NaCl, 2.5 mM KCl, 1 mM NaH_2_PO_4_, 26 mM NaHCO_3_, 1.3 mM MgCl_2_, 2.5 mM CaCl_2_, and 10 mM d-glucose) maintained at 31 °C. The ACSF-submerged slices were incubated 15–30 min before patching the cells.

Miniature AMPA receptor-mediated excitatory postsynaptic currents (mEPSCs) recordings were performed in the ASCF routinely containing picrotoxin (50 μM; Sigma, P1675) and TTX (0.5 μM; Tocris, 1078), to block GABA_A_ receptor-mediated currents and action potentials firing, respectively. NMDA receptor–mediated currents were blocked by Mg^2+^ supplemented in the extracellular solution, as AMPA mEPSCs were recorded at a holding potential of −60 mV. The AMPAR mEPSCs were recorded from pyramidal neurons, identified visually in the CA1 region of the hippocampal organotypic slice. The glass patch electrodes (3–6 MΩ resistance) were filled with intracellular solution (130 mM Cs-Gluconate, 20 mM HEPES, 0.4 mM EGTA, 4 mM QX-314Cl, 3 mM TEA-Cl, 4 mM Na_2_-ATP, 0.3 mM Na_2_-GTP, pH = 7.0–7.1, osmolarity: 290–295 mOsm). The patch electrodes were made from glass capillaries (OD: 1.2 mm, ID: 0.68 mm, WPI, 1B120F-4) and pulled using a Flaming-Brown micropipette puller (Sutter Instrument, P.1000).

For miniature inhibitory postsynaptic currents (mIPSCs) recordings from the CA1 pyramidal neurons, patch electrodes were filled with another intracellular solution (120 mM CsCl, 10 mM NaCl, 20 mM HEPES, 0.4 mM EGTA, 3 mM TEA-Cl, 4 mM Na_2_-ATP, 0.3 mM Na_2_-GTP, pH = 7.0–7.1, osmolarity: 290–295 mOsm). mIPSCs were recorded at a holding potential of −70 mV, and the ACSF was supplemented with TTX and NBQX (10 μM; Tocris, 1044) to block action potentials and AMPA receptor–mediated responses, respectively. At −70 mV membrane potential, the NMDA receptor–mediated responses were blocked due to the presence of Mg^2+^ in the extracellular solution.

Recorded raw data of miniature excitatory and inhibitory currents were acquired with Igor Pro 6.2 software. Using Mini Analysis software, the properties of miniature currents including amplitude (−pA) and interevent interval (s) were analyzed. At least 200 typical events were analyzed and presented on cumulative distribution plots and bar graphs representing the mean values.

To measure the paired-pulse ratio (PPR), the Schaffer collateral fibers were electrically stimulated by a flexible stimulus isolator (ISO-Flex, A.M.P.I.). Simultaneously, whole-cell recording of evoked AMPAR EPSCs was performed from a CA1 pyramidal cell. The ACSF was modified to contain 4 mM MgCl_2_ and 4 mM CaCl_2_, supplemented with picrotoxin (50 μM). Two electric stimuli triggering presynaptic action potentials were paired with increasing interstimulus intervals (50, 100, 200 ms). At least 50 stable AMPAR EPSCs responses for each interstimulus interval at a holding potential of −60 mV were recorded.

To investigate the change of synaptic strength, both AMPAR- and NMDAR-mediated EPSCs were recorded from the same CA1 neurons. The ACSF and intracellular solution were the same as the ones used for the PPR test. Evoked AMPAR EPSCs were recorded at a holding potential of −60 mV and, next, the holding potential was switched to +40 mV for recording NMDAR EPSCs. The amplitudes of NMDAR EPSCs were measured 50 ms after the peak, to ensure the lack of AMPAR component at +40 mV. For each recorded cell, the mean ratio of AMPAR/NMDAR amplitudes was calculated.

To evaluate the decay of NMDA receptors responses, whole-cell recordings of NMDAR EPSCs were conducted in the presence of NMDAR irreversible open-channel blocker MK-801 (20 μM; Sigma, M107) and AMPAR antagonist NBQX (10 μM; Hessler et al. 1993). The speed of the decay of NMDARs EPSCs amplitudes is directly proportional to the release probability. The Schaffer collateral fibers were electrically stimulated, and NMDAR EPSCs were recorded at a holding potential of +40 mV from a CA1 pyramidal cell. The recording was continued until NMDAR responses were no longer visible. The decay of amplitudes was calculated and presented as the percentage of the baseline amplitude.

In order to investigate the excitatory input onto GABAergic neurons, AMPA receptor-mediated mEPSCs were recorded from fast-spiking GABAergic neurons. To conduct this experiment, hippocampal organotypic slice cultures were prepared from GAD65-tdTomato transgenic mice, in which the inhibitory neurons are labeled with a red fluorescent protein tdTomato (Besser et al. 2015). The firing pattern of GAD65-tdTomato-positive neurons was determined using current-clamp recordings while injecting increasing currents (100–400 pA). The number of spikes was counted for each injected current. All cells, which fired 5–10 action potentials at 100 pA current injection and 25–30 spikes at 400 pA, were considered as fast firing. In such GABAergic neurons, the recording was switched to the voltage-clamp mode, ACSF replaced with one supplemented with TTX and AMPAR mEPSCs were recorded. The K-gluconate–based intracellular solution was used for recording miniature excitatory currents from GABAergic neurons (120 mM K-Gluconate, 2 mM MgCl_2_, 0.4 mM EGTA, 0.1 mM CaCl_2_, 10 mM HEPES, 2.5 mM Na_2_-ATP, 0.25 mM Na_2_-GTP, pH = 7.0–7.1, osmolarity: 290–295 mOsm).

#### Local Field Potentials Recording

In order to evaluate the rhythmic activity induced by Cch (10 μM) in organotypic slice cultures, local filed potentials recording was performed using HEKA Chartmaster software and ELC-03XS amplifier (npi). Recording electrodes (3–5 MΩ) filled with ACSF were positioned in the CA1 pyramidal layer. Ten minutes of baseline activity was recorded before application of Cch, and the next 1 h of field recording was conducted following the application of Cch. Recorded data were analyzed offline using a Spike-2.7 software computing system (Cambridge Electronic Design). 2.5 s samples of rhythmic activity were subjected to power/frequency (FFT) analysis.

### Gelatin Zymography

In order to evaluate the activity of MMP-9 released to the conditioned culture media, gelatin zymography technique was performed (modified from Szepesi et al. 2013). Modified culture medium used for this experiment contained B-27 supplements instead of horse serum and was exchanged 2–3 days before the day of collecting. Following Cch treatment, equal amounts of conditioned culture media were collected at 2, 4, 8, 16, and 24 h and kept in −80 °C until further analysis. A standard 8% sodium dodecyl sulfate-polyacrylamide gel electrophoresis (SDS-PAGE) gel in which the resolving gel contained 2 mg/mL gelatin was prepared. The samples (culture medium) were mixed with the nonreducing SDS sample buffer (Bio-Rad) and incubated for 10 min at room temperature. Next, they were loaded onto the SDS-PAGE gel and the electrophoresis was conducted at constant voltage (120 V) for 60–90 min. The gel was removed from electrophoresis apparatus and washed two times (each 30 min) with 2.5% Triton-X-100. After that, it was incubated in developing buffer (50 mM Tris-Cl pH = 7.5; 10 mM CaCl_2_; 1 μM ZnCl_2_; 1% Triton-X-100; 0.02% NaN_3_) with gentle shaking at 37 C for 1 week, allowing for MMP-9 renaturation and cleaving the gelatin present in the gel after SDS-PAGE. Finally, the gel was stained with Coomassie blue solution for 20 min and washed until MMP-9 and MMP-2 activity-related clear bands appeared on the gel. The gels were scanned and the intensity of MMP-9 and MMP-2 activity was measured with GeneTools software, Syngene. Due to the medium change with a fresh one (at 1 h) to wash out the Cch, the intensity of active MMP-9 measured at 2 and 4 h was very low and not sufficient to be properly recognized by the scanning software. Thus, raw data of the active MMP-9 bands’ intensities were presented only for 8, 16, and 24 h.

### Western Blot

With the aim of measuring the expression of MMP-9 and TIMP-1 on protein level, immunoblotting was performed. Due to the lower level of detection of MMP-9 in WB comparing to gelatin zymography and to avoid albumin overload in samples, experiments with Cch stimulation were performed in ACSF (the same formulation as for electrophysiological studies) in order to concentrate proteins (Frankowski et al. 2012). Following Cch treatment, equal amounts of conditioned ACSF were collected at 2, 4, 8, 16, and 24 h and kept in −80 °C until further analysis. The proteins from the ACSF were precipitated in 60% ethanol for 1 min at 4 C and then centrifuged at 15 000*g* for 5 min (Szklarczyk et al. 2002). The precipitates were solubilized in 7× smaller volume of the SDS sample buffer (Bio-Rad) mixed with Bond Breaker (Thermo Fisher Scientific). Subsequently, the samples were incubated for 6 min at 96 C. Proteins were separated by SDS-PAGE with standard 8% gel and transferred to polyvinylidene difluoride membranes (Immobilon-P, Bio-Rad; 200 mA). The membranes were blocked (1 h, RT), incubated with primary antibody (MMP-9: 1:1000, catalog no. AB19016, Sigma-Aldrich; TIMP-1: 1:500, catalog no. MAB580, R&D Systems) overnight (4 °C), washed 3×in Tris-buffered saline/0.1% Tween-20 (TBS-T; 10 min, shaking), and incubated with secondary HRP-conjugated antibodies (in blocking buffer, 1 h, RT; anti-rabbit P-100, 1:5000; anti-mouse P-200, 1:5000; Vector Laboratories). After TBS-T washing, detection of the immunoreaction was performed with an enhanced chemiluminescence (ECL) kit (Amersham BioSciences) and detected with a use of ChemiDoc MP Imaging System (Bio-Rad).

### In Situ Zymography

Organotypic hippocampal slices were stimulated with 10 μM Cch solution. Culture media was replaced for fresh media with 0.1% v/v DMSO. After 30 minutes, 2 μL of 5 mM fresh Cch solution or distilled water was added to the culture media. Slices were incubated for 1 h, washed with fresh media containing 0.1% v/v DMSO, and incubated for additional 7 or 15 h. After the incubation, all cultures were washed with CO_2_ buffered ACSF. Dye-quenched (DQ) gelatin (40 μg/mL), dissolved in CO_2_ buffered ACSF, was poured on the surface of the slices and incubated in 37 C in 5% CO_2_. After 1 h of incubation, DQ gelatin was gently washed off with PBS and the slices were fixed in 4% paraformaldehyde (PFA) with 4% sucrose solution for 20 minutes in room temperature, followed by three times washing with PBS supplemented with 4% sucrose. Membranes with hippocampal slices were cut out from the inserts and mounted with DAPI Fluoromount-G (SouthernBiotech) and covered with the coverslip.

### Immunocytochemistry

Organotypic slice cultures were fixed with 4% PFA during overnight incubation at 4 °C (Gogolla et al. 2006a, 2006b). The PFA was washed with PBS three times for 30 min. The fixed slices were treated with 0.1% glycine for 15 min and permeabilized using PBS containing 0.5% Triton-X-100 (PBST) for 3 h with gentle shaking at room temperature. Next, the slices were blocked by 10% bovine serum albumin (BSA)/10% normal goat serum (NGS) in 0.25% PBST with gentle shaking at 4 C overnight. Then, they were incubated with primary antibodies diluted in 5% BSA/5% NGS in 0.1% PBST with gentle shaking at 4 C overnight. The primary antibodies were washed with 0.1% PBST four times for 30 min. Two primary antibodies against VGLUT-1 (Synaptic Systems; 135 511) and PSD-95 (Invitrogen; 51-6900) were used. The final concentration of VGLUT-1 antibody was 1/500 and PSD-95 was adjusted to 1/200. After that, the slices were incubated with appropriate secondary antibodies diluted in 0.1% PBST (1/200) for 2 h with gentle shaking at room temperature. The secondary antibodies were washed with 0.1% PBST four times for 30 min. The slices were finally transferred on the glass slides, mounted with DAPI Fluoromount-G, and covered with the coverslip. The slides were stored at 4 C until imaged with confocal microscope.

### Biocytin Labeling

The number of dendritic protrusions was analyzed by labeling the CA1 pyramidal neuron with biocytin (Sigma; B4261; Wilson and Sachdev 2004). To conduct this experiment, the organotypic slices were transferred to the electrophysiology chamber and the CA1 pyramidal neuron was patched and filled with the intracellular solution supplemented with 0.5% biocytin. After 15–20 min allowing the biocytin to fill the cell body and its dendrites, the glass electrode was gently removed and the whole slice was immediately transferred to 4% PFA and kept overnight at 4 C. The fixed slices were washed with 0.1% PBST three times for 30 min. Next, they were incubated with Alexa-Fluor–conjugated streptavidin diluted in 0.1% PBST (1/200) for 2 h at room temperature. Then, the slices were washed with 0.1% PBST three times for 30 min and transferred on the glass slides and mounted with DAPI Fluoromount-G and covered with the coverslip.

### Microscopy and Analysis

In situ zymography slices were imaged on ZEISS spinning DISC microscope with 10x magnification to select regions of interest (ROI, five regions from each hippocampal slice). The identified ROIs were imaged with 63× objective z-stack from the surface to 10 μm in depth with 1 μm steps.

The confocal microscope Zeiss LSM 780 was used to collect the images from all immunostained and biocytin-labeled slices. In case of the double immunolabeling of VGLUT-1 and PSD-95, confocal images were collected from four areas of hippocampal CA1 including pyramidal and stratum radiatum layers with the same configuration of microscope for control and test groups. The number and size of VGLUT-1– and PSD-95–positive puncta were analyzed by ImageJ software. The configuration of software including size (minimum & maximum), threshold, and brightness was the same for control and test groups. To analyze dendritic protrusions, z-stack confocal images of dendrites located in stratum radiatum were gathered. To eliminate possible systemic differences between dendrites of different ranks, the analysis was performed on secondary and tertiary apical dendritic branches. The ImageJ software was used to analyze the number of dendritic protrusions. Using the software, a part of dendrite was selected, in which the number of dendritic protrusions was manually counted.

### Statistical Analyses

Statistical analyses of cumulative distributions of miniature excitatory and inhibitory currents were performed using Kolmogorov-Smirnov test, and their mean data using nested one-way ANOVA test followed by Sidak’s multiple comparison test with GraphPad Prism 8.0 software. The mean data of dendritic protrusions density and AMPA/NMDA ratio were analyzed using Student’s *t*-test. The density and surface of VGLUT-1– and PSD-95–positive puncta, as well as the PPR test, were analyzed using nested one-way ANOVA test followed by Tukey’s multiple comparison test. The level of active MMP-9 from gelatin zymography was analyzed using mixed-effects repeated measures (RM) ANOVA (restricted maximum likelihood method) followed by Sidak’s multiple comparisons tests. The significance levels are indicated by asterisks: ^*^P < 0.05, ^*^^*^P < 0.01, ^*^^*^^*^P < 0.001.

## Results

### Cholinergic Stimulation Induces the Activity of MMP

**Figure 1:**
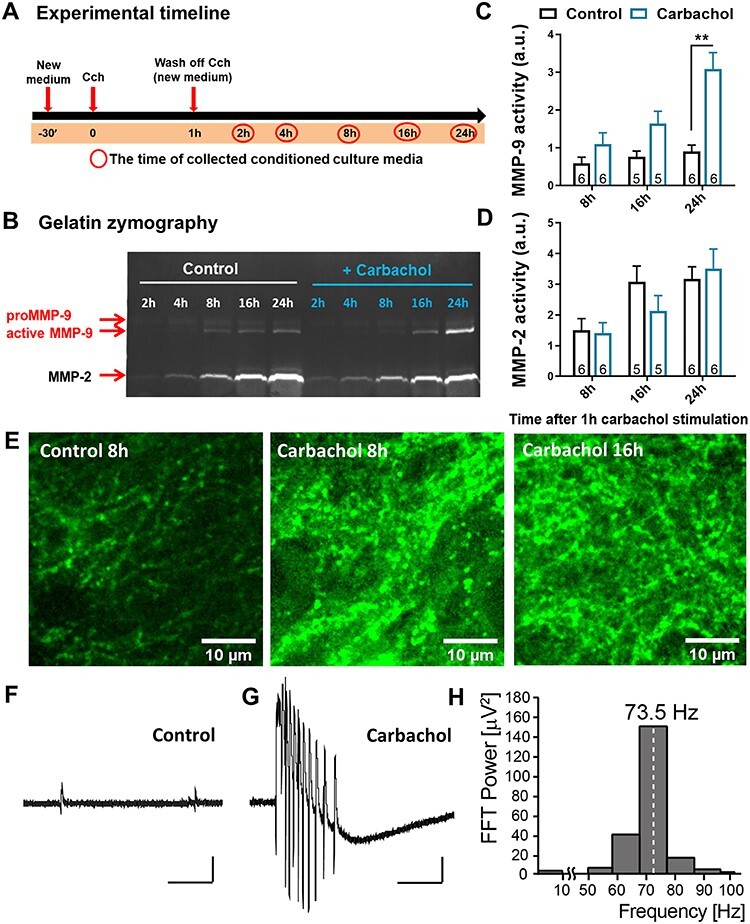
MMP-9 enzymatic activity timeline in the CA1 stratum radiatum area. (*A*) An experimental timeline representing different time points (red circles) of collecting conditioned culture media after 1 h of Cch (10 μM) treatment from hippocampal organotypic slice cultures. (*B*) An example of gelatin zymography, which shows the enzymatic activity of MMP-9 and MMP-2 in equal amounts of culture media from different time points up to 24 h after Cch, displayed in part A. Two forms of MMP-9, pro- and active enzyme, were detected based on the molecular weight. (*C*) Quantified data indicate that MMP-9 active form gradually increases after Cch treatment with a significant peak at 24 h (*F*_1,28_ = 26.62, *P* = 0.009, *n* = 6). (*D*) No difference is observed in MMP-2 activity. Data are represented as mean ± SEM; the values on bars represent the numbers of independent cultures). (*E*) Example confocal images of the intensity of DQ gelatin fluorescence 8 h after DMSO the (Ctrl, left), 8 h after Cch (middle) and 16 h after Cch treatment (right). (*F*) A field potential example trace representing a baseline activity of an organotypic slice. (*G*) A field potential recording of a burst of gamma oscillations during 1 h application of Cch. Scale bars: 200 ms; 10 μV. (*H*) Fast Fourier transform analysis of a gamma trace presented in *G*. Statistical comparisons were performed using mixed-effects RM ANOVA followed by Sidak’s multiple comparisons test (*P* value: ^*^^*^  *P* < 0.01).

To study the long-term synaptic plasticity in hippocampal organotypic slice cultures, we applied Cch (10 μM, 1 h)—a cholinergic agonist that in the hippocampus induces a robust potentiation of rhythmic activity (Huerta and Lisman 1993; De Roo et al. 2008). Such activity in theta and gamma range is essential in the hippocampus for episodic memory formation (Nyhus and Curran 2010). Thus, we perfused hippocampal organotypic cultures with Cch for 1 h and measured its effect on hippocampal rhythmic activity. We recorded local field potentials in the CA1 region of organotypic slice cultures and confirmed that Cch evokes oscillations in the gamma range (Fig. 1*F*–*H*). We then examined the effect of cholinergically induced gamma oscillations on the enzymatic activity of MMPs (Fig. 1*B*–*E*). Using gelatin zymography, we measured the enzymatic activity of two gelatinases—MMP-9 and MMP-2 in equal volumes of conditioned culture media, collected from slice cultures after Cch stimulation (at 2, 4, 8, 16 and 24 h, Fig. 1*A*,*B*). The zymography gels show a gradual accumulation of MMP activity in the cultured media (Fig. 1*B*). The quantification of zymography gels revealed a specific increase of the MMP-9 activity after Cch stimulation, with no change in MMP-2 activity, as compared to control-treated slices (MMP-9 24 h Ctrl: 0.9 ± 0.2 a.u., *n* = 6 cultures; Cch: 3.1 ± 0.4 a.u., *n* = 6 cultures, *F*_1,28_ = 26.62 *P* = 0.009, mixed-effects RM ANOVA followed by Sidak’s test). To verify whether this gradual increase of MMP-9 in gel zymography indeed reflects the increase in MMP-9 activity, we performed immunoblot analysis of its natural inhibitor tissue inhibitor of matrix metalloproteinases-1 (TIMP-1). We discovered a transient increase in TIMP-1 protein level at 16 h after Cch stimulation (Supplementary Fig. 1*C*). This increase coincided with elevated pro-MMP-9 protein at the same time point (Supplementary Fig. 1*B*). Notably, gel zymography demonstrated that specifically at 16 h post-Cch pro-MMP-9 form was the most markedly accumulated (Supplementary Fig. 1*A*).

To examine the localization of MMP action, we performed in situ zymography on hippocampal organotypic cultures. To this end, the slice cultures were treated with Cch (10 μM) for 1 h. After washing out the carbachol, the slices were incubated in the presence of DMSO for the remaining hours. In situ zymography revealed that the gelatinolytic activity of MMPs, measured as the intensity of the fluorescence of DQ gelatin, increases hours after the cholinergic stimulation (Fig. 1*E*). Of note, the gelatinolytic activity exhibits a punctuated pattern along the dendrites, suggestive of its localization at the dendritic spines, known to harbor postsynaptic domains of excitatory synapses. The size of the observed puncta as well as the pattern of enzymatic activity is a typical feature of MMP-9, in contrast to another gelatinase produced in such cultures, that is, MMP-2.

### Either Chemical Inhibition or Genetic Ablation of MMP-9 Enhances the Carbachol-Induced Potentiation of mEPSCs

**Figure 2:**
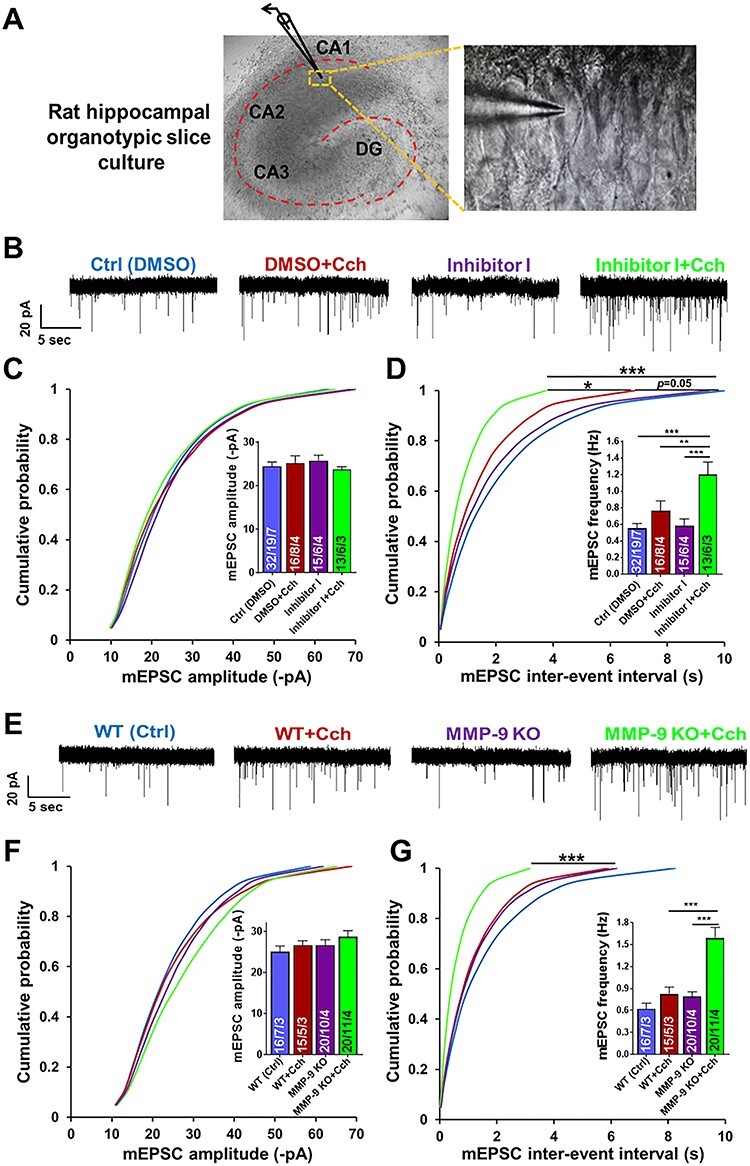
Chemical inhibition or genetic ablation of MMP-9 results in a dramatic increase of the frequency of AMPAR mEPSCs induced by Cch at hippocampal CA1 excitatory synapses. (*A*) A DIC image of rat hippocampal organotypic slice culture displaying a patched pyramidal cell in the CA1 region. (*B*) Representative traces of mEPSCs from different experimental groups. (*C*) Cumulative distributions of event amplitudes and mean event amplitudes (inset) display no effect of Cch (10 μM) on mEPSCs amplitude. (*D*) Cumulative distributions of interevent intervals and mean event frequencies (inset) show an increase of mEPSCs frequency after Cch and further increase by inhibition of MMP-9 activity. (*E*) Representative traces of mEPSCs. (*F*) Cumulative distributions of event amplitudes and mean event amplitudes (inset) show no difference of AMPAR mEPSCs amplitude between WT or MMP-9 KO slices, control and Cch treated (mouse hippocampal organotypic slice cultures). (*G*) Cumulative distributions of interevent intervals and mean event frequencies (inset) of MMP-9 KO slices reveal a dramatic increase of AMPAR mEPSCs frequency after Cch stimulation compared to KO and WT controls. Statistical comparisons on cumulative distributions were performed with Kolmogorov-Smirnov test and nested one-way ANOVA followed by Sidak’s test for bar graph inserts (mEPSCs frequency: *F*_3,34_ = 12.70, Inh I + Cch vs. Ctrl *P* < 0.001, Inh I + Cch vs. Cch *P* = 0.007, Inh I + Cch vs. Inh I *P* = 0.0002; mEPSCs frequency in MMP-9 KO: *F*_3,29_ = 17.04, MMP-9 KO + Cch vs. WT + Cch *P* = 0.0003, MMP-9 KO + Cch vs. MMP-9 KO *P* < 0.0001). Numbers inside each bar represent the numbers of neurons/slices/cultures. The number of slices was used for statistical analysis (*P* value: ^*^P < 0.05, ^*^^*^P < 0.01, ^*^^*^^*^P < 0.001).

To test the consequences of the cholinergic stimulation and the potentiation of MMP-9 activity on the excitatory transmission in organotypic slices, we performed electrophysiological recordings of miniature excitatory postsynaptic currents (mEPSCs) from pyramidal neurons in the CA1 region. We examined the long-term effect of Cch stimulation on pyramidal neurons and the requirement of carbachol-induced increase in MMP-9 activity in this plasticity process. Organotypic cultures were perfused either with DMSO or with MMP-9 Inhibitor I (5 μM), followed by Cch stimulation for 1 h. After Cch washout, the slices were again incubated in the presence of DMSO or the Inhibitor I for the remaining time. We measured AMPAR-mediated mEPSCs and recordings were performed within 14–22 h after Cch stimulation. We discovered that Cch alone potentiated the mEPSCs frequency (Ctrl: 0.55 ± 0.06 Hz, *n* = 19 slices; Cch: 0.75 ± 0.12 Hz *n* = 8, *P* = 0.05, KS test; Fig. 2*D*), but MMP-9 inhibition further enhanced this phenomenon, causing over 50% increase in mEPSCs frequency (1.20 ± 0.15 Hz, *n* = 6, *P* < 0.001, KS test). This effect was specific to Cch-induced plasticity, as MMP-9 inhibitor alone did not alter the frequency of currents (0.58 ± 0.07 Hz, *n* = 6, *P* = 0.5 vs. Ctrl, KS test). No differences in the amplitudes of mEPSCs were observed (Ctrl: −24.5 ± 1.0 pA, *n* = 19; Cch: −25.3 ± 1.6 pA, *n* = 8; Inhibitor I: −25.8 ± 1.2 pA, *n* = 6; Inhibitor I + Cch: −23.8 ± 0.6 pA, *n* = 6; Fig. 2*C*).

As Inhibitor I, although being a very potent MMP-9 blocker, affects the activity of other metalloproteinases as well, we confirmed the involvement of MMP-9 activity in Cch-induced plasticity by using organotypic slices obtained from MMP-9 knockout (KO) mice (Vu et al. 1998). Similar to the chemical manipulation of MMP-9 activity, Cch did not affect events amplitude in MMP-9 KO slices (WT: −25.0 ± 1.4 pA, *n* = 7; WT + Cch: −26.7 ± 1.1 pA, *n* = 5; KO: −26.7 ± 1.3 pA, *n* = 10; KO + Cch: −28.7 ± 1.5 pA, *n* = 11 slices; Fig. 2*F*). However, the MMP-9 KOs showed a significant enhancement of the frequency of AMPAR mEPSCs after Cch induction (1.59 ± 0.15 Hz, *n* = 11) compared to the KO ctrl slices (0.79 ± 0.07 Hz, *n* = 10, *P* < 0.001, KS test; Fig. 2*G*). Without Cch, the frequency of mEPSCs in MMP-9 KO slices was not different from WT (WT: 0.62 ± 0.08 Hz, *n* = 7, *P* = 0.6, KS test). Thus, genetic ablation of MMP-9 intensified the action of Cch by elevating the frequency of AMPA receptor–mediated mEPSCs, similar to the inhibition of MMP-9 activity (Fig. 2*D*). This experiment confirms that the dramatic increase in the frequency of minievents at hippocampal CA1 synapses is specific to the inhibition of MMP-9 activity.

### Carbachol-Mediated Increase in the Inhibitory Synaptic Transmission in the Hippocampus is Impaired by Blockage of MMP-9

**Figure 3:**
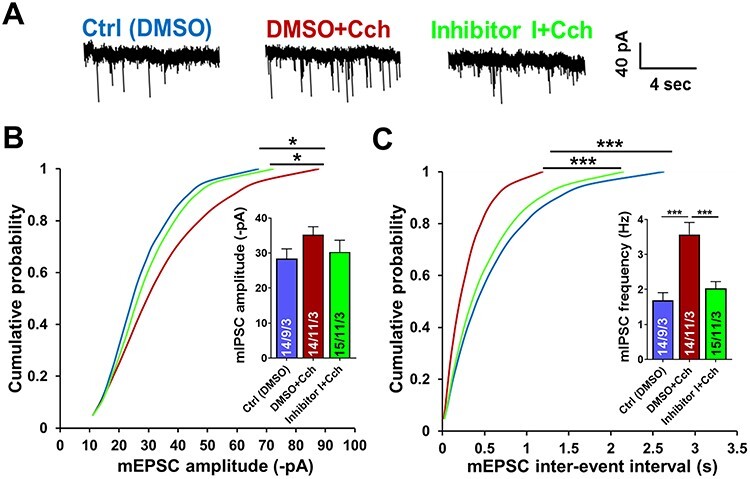
MMP-9 blocking impairs cholinergic activation-mediated increase of inhibitory input onto hippocampal CA1 pyramidal neurons. (*A*) Representative traces of mIPSCs from different experimental groups. (*B*) Cumulative distributions of event amplitudes and mean event amplitudes (inset) display an increase in mIPSCs amplitude by Cch, and a slight impairment by inhibition of MMP-9. (*C*) Cumulative distributions of interevent intervals and mean event frequencies (inset) show almost 2-fold enhancement of inhibitory currents frequency by Cch that is disrupted by blockage of MMP-9. Data shown as mean ± SEM (numbers of neurons/independent cultures). Statistical comparisons on cumulative distributions were performed using Kolmogorov-Smirnov test and nested one-way ANOVA followed by Sidak’s test for bar graph inserts (mIPSCs frequency: *F*_2,28_ = 14.06, Cch vs. Ctrl *P* = 0.0001, Inh I + Cch vs. Cch *P* = 0.0005). Numbers inside each bar represent the numbers of neurons/slices/cultures. Statistical analysis was based on the number of slices (*P* value: ^*^P < 0.05, ^*^^*^^*^P < 0.001).

It is likely that application of Cch in micromolar concentrations could lead to the inhibition of synaptic transmission in the hippocampus (De Roo et al. 2008; Drever et al. 2011). Thus, the increase in excitatory events frequency could be the result of diminished inhibitory transmission within the hippocampal formation. Therefore, we looked into the inhibitory synaptic transmission several hours following 1 h of 10 μM Cch treatment and measured mIPSCs from the CA1 neurons. We found that inhibition of MMP-9 significantly disrupted Cch-mediated enhancement of the frequency of miniature inhibitory currents from 3.57 ± 0.34 to 2.03 ± 0.20 Hz (*n* = 11 slices and *n* = 9 slices, respectively, *P* < 0.001, KS test; Fig. 3*C*). We also observed an increase of mIPSCs amplitude (Ctrl: −28.4 ± 2.8 pA, *n* = 9; Cch: −35.2 ± 2.3 pA, *n* = 11, *P* = 0.01, KS test), which was not present when the MMP-9 activity was blocked (Inhibitor I + Cch: −30.3 ± 3.3 pA, *n* = 11, *P* vs. Cch = 0.01, KS test; Fig. 3*B*). Thus, MMP-9 activity is necessary to mediate cholinergically induced potentiation of the inhibitory input onto CA1 pyramidal cells.

### M‌MP-9 Blocking Prevents Carbachol-Mediated Increase in the Excitatory Input onto a Subpopulation of Fast-Spiking GABAergic Neurons

**Figure 4:**
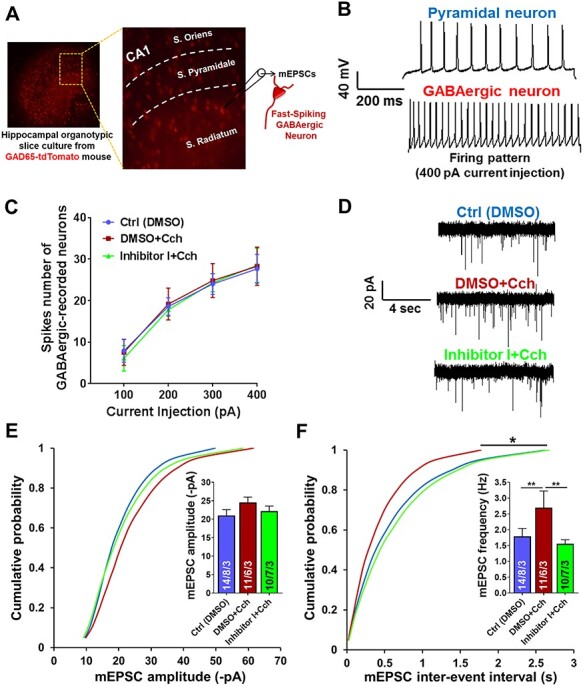
Blockage of MMP-9 prevents the enhancement of excitatory input onto a subpopulation of fast-spiking GABAergic neurons, mediated by cholinergic activation. (*A*) Hippocampal organotypic slice culture from GAD65-tdTomato mouse. AMPAR mEPSCs were recorded from stratum radiatum fast-spiking GAD65-positive neurons. (*B*) Examples of APs traces of the CA1 pyramidal neuron and the fast-spiking GABAergic neuron induced by 400 pA current injection. (*C*) Spikes number of GABAergic neurons, subjected to record AMPAR mEPSCs, demonstrates the same firing pattern induced by different current injections (100, 200, 300, 400 pA). (*D*) Representative traces of AMPAR mEPSCs. (*E*) Cumulative distributions of event amplitudes and mean event amplitudes (inset) indicate no difference in the amplitudes. (*F*) Cumulative distributions of interevent intervals and mean event frequencies (inset) reveal significant increase in the frequency of miniature excitatory input onto the fast-spiking GABAergic neurons by Cch and its disruption when MMP-9 is blocked. Data shown as mean ± SEM (numbers of neurons/independent cultures). Statistical comparisons on cumulative distributions were performed using Kolmogorov-Smirnov test and nested one-way ANOVA followed by Sidak’s test for bar graph inserts (mEPSCs frequency: *F*_2,32_ = 6.52, Cch vs. Ctrl *P* = 0.009, Inh I + Cch vs. Cch *P* = 0.009). Numbers inside each bar represent the numbers of neurons/slices/cultures. Statistical analysis was based on the number of slices (*P* value: ^*^P < 0.05, ^*^^*^P < 0.01).

Thus far, we discovered that cholinergic stimulation potentiates excitatory and inhibitory currents on CA1 pyramidal cells. This effect is MMP-9 dependent, as blocking MMP-9 activity prevents inhibitory inputs potentiation, which, in turn, leads to the dramatic enhancement of the frequency of the excitatory mEPSCs. Therefore, we asked whether MMP-9 inhibition affects GABAergic inhibitory neurons. To address this question, we prepared organotypic cultures from a transgenic mouse line expressing the red fluorescent protein tdTomato in the inhibitory, GAD65-positive neurons (Fig. 4*A*; Besser et al. 2015). To record AMPAR mEPSCs in the presence of the MMP-9 blocker, we searched the CA1 stratum radiatum for the fast-spiking GAD65-positive neurons with similar firing pattern. This ensures that selected GAD65-positive neurons from all experimental groups belong to the same population, at least based on firing patterns, as inhibitory neurons can be highly heterogeneous in their function and activity.

The firing patterns were defined by the number of spikes generated by current injections from 100 to 400 pA. Thus, inhibitory neurons, which were selected for AMPAR mEPSCs analysis, were characterized by similar firing patterns (Fig. 4*B*,*C*). Contrary to the results from CA1 neurons, recordings of mEPSCs demonstrated that inhibition of MMP-9 activity prevented the enhancement of mEPSCs frequency in the fast-spiking GABAergic neurons (Ctrl: 1.79 ± 0.25 Hz, *n* = 8 slices, Cch: 2.69 ± 0.52 Hz, *n* = 6; Inh I + Cch: 1.55 ± 0.13 Hz, *n* = 7, *P* vs. Cch = 0.01, KS test; Fig. 4*F*). The Inhibitor I–induced reduction in mEPSCs frequency suggests that MMP-9 activity is crucial for Cch-mediated potentiation of fast-spiking inhibitory neurons by facilitating their excitatory currents input.

### Cholinergic Muscarinic Receptors Mediate the Enhancement of AMPAR mEPSCs Frequency

**Figure 5:**
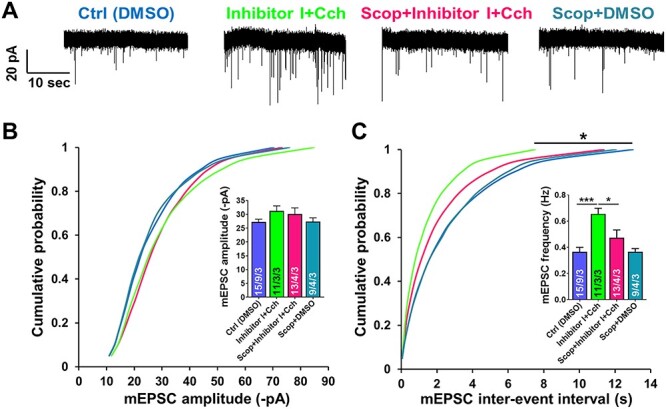
Muscarinic receptors–mediated cholinergic activation mediates enhancement of AMPAR mEPSCs frequency at hippocampal CA1 excitatory synapses. (*A*) Representative traces of AMPAR mEPSCs. (*B*) Cumulative distributions of event amplitudes and mean event amplitudes (inset) display no significant difference in mEPSCs amplitude. (*C*) Cumulative distributions of interevent intervals and mean event frequencies (inset) demonstrate that enhancement of AMPAR mEPSCs frequency, which is mediated by MMP-9 inhibition and Cch, is impaired by cholinergic muscarinic receptors antagonist (Scop). Data shown as mean ± SEM (numbers of neurons/independent cultures). Statistical comparisons were performed using Kolmogorov-Smirnov test on cumulative distributions and nested one-way ANOVA followed by Sidak’s test for bar graph inserts (mEPSCs frequency: *F*_3,16_ = 10.26, Inh I + Cch vs. Ctrl *P* = 0.0009, Scop + Inh I + Cch vs. Inh I + Cch *P* = 0.01). Numbers inside each bar represent the numbers of neurons/slices/cultures. Statistical analysis was based on the number of slices (*P* value: ^*^P < 0.05, ^*^^*^^*^P < 0.001).

In order to identify which type of cholinergic receptors mediates the mEPSCs frequency potentiation, we applied a muscarinic receptor antagonist scopolamine (Scop, 30 μM). We recorded AMPAR mEPSCs in the presence of Scop, to define whether the increased frequency of miniature currents evoked by Cch is mediated by muscarinic receptors (Fig. 5). Event frequency analysis revealed a significant increase in the incidence of AMPAR mEPSCs after Cch when combined with the MMP-9 blocker (0.66 ± 0.04 Hz, *n* = 3 slices) compared to control (0.36 ± 0.03 Hz, *n* = 9, *P* < 0.03, KS test). Moreover, this increase was not present when slices were pretreated with Scop (0.47 ± 0.06 Hz, *n* = 4, *P* = 0.3, KS test; Fig. 5*C*). The frequency of mEPSCs in slices treated only with Scop did not differ from control (0.37 ± 0.02 Hz, *n* = 4, *P* = 0.7, KS test).

### Enhancement of the Frequency of AMPAR mEPSCs is Not a Result of the Increased Release Probability

As described above, our results indicated an enhancement of the frequency of miniature excitatory currents following Cch-induced plasticity in the CA1 principal neurons. This increase was further boosted by blocking MMP-9 activity. Thus, we set out to look into a possible cellular mechanism that triggers this enhancement of minievents frequency. We examined two putative major phenomena: a higher presynaptic release probability or an increase in the number of excitatory synapses.

**Figure 6:**
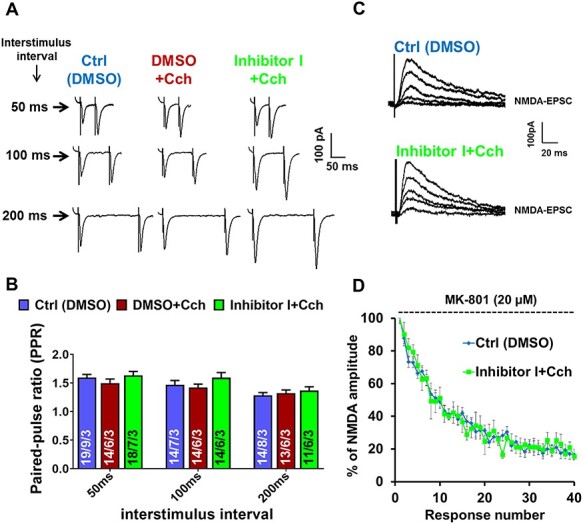
Carbachol does not affect the probability of glutamate release. (*A*) Example of PPR traces of AMPA receptor–mediated EPSCs with three interstimulus intervals (50, 100, 200 ms) from different experimental groups. (*B*) Quantified data of PPR of amplitudes from stimulated groups (Cch and Inhibitor I + Cch) reveal no significant difference compared to control in neither of three interstimulus intervals. (*C*) Example traces of consecutive NMDAR responses in the presence of open-channel blocker MK-801. (*D*) Decay of NMDA receptor EPSCs amplitude normalized to the first amplitude recorded in the presence of MK-801 showing that inhibition of MMP-9 does not affect the decay kinetics of NMDAR responses (*n* = 6 neurons). Data in *B* are represented as mean ± SEM. Numbers inside each bar represent the numbers of neurons/slices/cultures. Statistical analysis was based on the number of slices.

The probability of the presynaptic release was first assessed using the PPR test. The PPR is inversely correlated with the probability of neurotransmitter release at synapses (Dobrunz and Stevens 1997). To perform the PPR test, a subset of the Schaffer collateral pathway was electrically stimulated using two electric pulses with increasing interstimulus intervals (50, 100, and 200 ms). The amplitudes of these paired AMPAR EPSCs from the CA1 pyramidal neurons were recorded and the ratio of the second to the first postsynaptic response (*R* = P2/P1) was calculated. The analysis revealed no significant differences between the experimental groups (e.g., for 50 ms interstimulus interval PPR: Ctrl: 1.6 ± 0.05; Cch: 1.5 ± 0.07; Inhibitor I + Cch: 1.6 ± 0.07, *F*_2,19_ = 0.9, Cch *P* = 0.5, Cch + Inh I *P* = 0.8, nested one-way ANOVA test; Fig. 6*B*).

It has been shown that PPR can be affected by AMPA receptor desensitization and lateral diffusion (Heine et al. 2008; Constals et al. 2015). The rapid exchange of desensitized AMPA receptors by naive ones is crucial in maintaining a high-frequency synaptic transmission, which takes place in the hippocampus. Therefore, we conducted an alternative electrophysiological test, in which MK-801, an irreversible NMDA receptor open-channel blocker, was used to assess AMPAR-independent release probability (Hessler et al. 1993; Rosenmund et al. 1993). Evoked NMDAR EPSCs were recorded via the Schaffer collateral pathway stimulation. The speedup of MK-801 use-dependent decay of NMDAR EPSCs amplitudes would indicate the increased presynaptic release. However, no changes in the decay of NMDARs amplitudes were detected, neither after Cch induction nor after MMP-9 inhibition (Fig. 6*D*). Thus, we conclude that the cholinergically induced enhancement of mEPSCs frequency could not be the result of the increase in the glutamate release probability.

### Enhancement of the mEPSCs Frequency is Caused by the Increase in the Number of Excitatory Synapses

**Figure 7:**
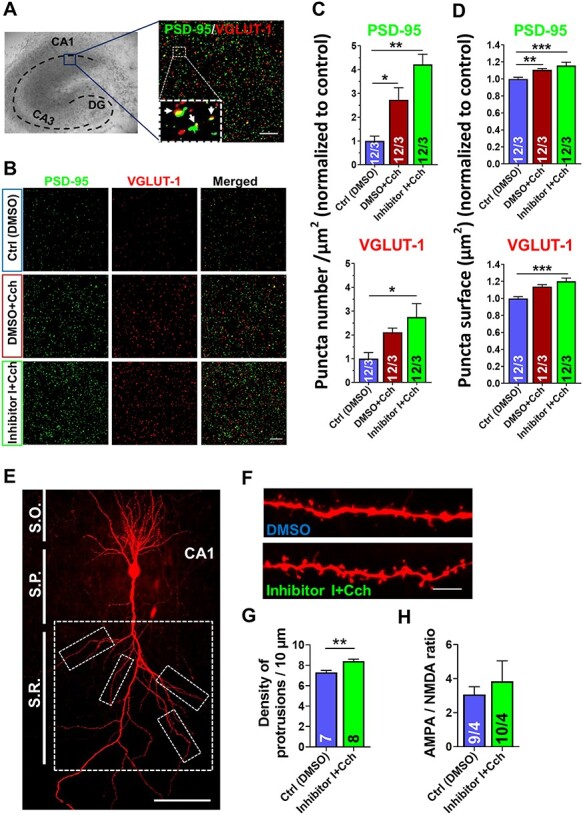
Augmented excitatory synaptogenesis and increase in dendritic protrusions density mediated by cholinergic activation is boosted by inhibition of MMP-9. (*A*) An illustration of rat hippocampal organotypic slice culture where blue box indicates the region imaged with confocal microscope, and an example of immunolabeling of PSD-95 (green) and VGLUT-1 (red; merged image), with their colocalizations shown with arrows in the magnified box. Scale bars: 10 μm. (*B*) Representative examples of double immunolabeling of PSD-95 and VGLUT-1 from experimental groups. Scale bars: 10 μm. Quantitative measurement of PSD-95– or VGLUT-1–positive puncta reveals a significant increase in the number (*C*) as well as surface area (*D*) 21 h after Cch, normalized to control, with further enhancement in the presence of MMP-9 blocker (*n* = 48 images; 12 slices; 3 independent cultures). Forty-four images of PSD-95 puncta were analyzed in the control (DMSO) group. (*E*) A CA1 pyramidal neuron filled with biocytin and stained with Alexa-Fluor–conjugated streptavidin. Dashed boxes represent areas on secondary and tertiary apical dendrites where protrusions density was measured. Scale bar: 100 μm. S.R.—stratum radiatum, S.P.—stratum pyramidale, S.O.—stratum oriens. (*F*) Examples of secondary apical dendrites. Scale bars: 5 μm. (*G*) Dendritic protrusions analysis shows a significant density increase in the Inhibitor I + Cch group (*n* = 8 pyramidal neurons (8 slices)/56 dendritic segments) compared to control (DMSO, *n* = 7 pyramidal neurons (7 slices)/48 dendritic segments). (*H*) The ratio of AMPA/NMDA receptors–mediated EPSCs amplitudes in CA1 pyramidal neurons shows no significant difference between Ctrl and Inh I + Cch (*n* = 4 slices), (numbers inside each bar represent the numbers of neurons/slices). Data shown as mean ± SEM. Statistical comparisons of puncta number and surface were based on the number of independent cultures and performed with nested one-way ANOVA test followed by Tukey’s multiple comparison test. Statistical analysis of protrusions density was based on the number of slices and performed with Student’s *t*-test (*P* value: ^*^P < 0.05; ^*^^*^P < 0.01; ^*^^*^^*^P < 0.001).

To visualize excitatory synapses on the CA1 neurons, a double immunostaining of the postsynaptic density protein 95 (PSD-95), a marker for mature dendritic spines, and the vesicular glutamate transporter 1 (VGLUT-1), a marker for functional glutamatergic presynaptic terminals, was performed (Fig. 7*A*). Hippocampal organotypic slices were subjected to double-immunolabeling of PSD-95 and VGLUT-1, 21 h after Cch treatment. Four regions of the CA1 area of each slice were imaged. Density and surface area of PSD-95– or VGLUT-1–positive puncta were measured for each individual image. Data were normalized to the control (DMSO) from each repetition. We observed a significant increase in the number (PSD-95: 2.7 ± 0.2/μm^2^, *F*_2,6_ = 17.20, *P* = 0.045, nested one-way ANOVA; Fig. 7*C*) as well as a small increase in surface area (PSD-95: 1.1 ± 0.01 μm^2^, *F*_2,6_ = 26.71, *P* = 0.007 (Fig. 7*D*) of PSD-95–positive puncta after Cch induction. A further enhancement of the number (PSD-95: 4.2 ± 0.2/μm^2^, *F*_2,6_ = 17.20, *P* = 0.003; VGLUT-1: 2.7 ± 0.1/μm^2^, *F*_2,6_ = 6.65, *P* = 0.03; Fig. 7*C*) and surface area (PSD-95: 1.16 ± 0.01 μm^2^, *F*_2,6_ = 26.71, *P* = 0.0009; VGLUT-1: 1.20 ± 0.2 μm^2^, *F*_2,6_ = 3.75, *P* = 0.0009; Fig. 7*D*) of both mentioned markers was observed when Cch was combined with the MMP-9 blocker.

Following the observation of De Roo et al. (2008) that Cch increases dendritic spine turnover in CA1 pyramidal neurons, we assessed the density of dendritic protrusions. Z-Stacks confocal microscopy images were collected from the secondary apical dendritic segments of the CA1 pyramidal cells, which had been patched and filled with biocytin (Fig. 7*E*,*F*). The results showed a significant increase of dendritic protrusion density in the Inhibitor I + Cch group (8.4 ± 0.2/10 μm, *n* = 7 neurons, 14–21 h after Cch stimulation) compared to the control (7.3 ± 0.2/10 μm, *n* = 8 neurons, *P* = 0.002, *t*-test; Fig. 7*G*). Taken together, these data demonstrate Cch-induced increase in the number of excitatory synapses, which is further increased by blocking MMP-9 activity.

The increase in the surface area of PSD-95–positive puncta (Fig. 7*D*) is suggestive of the increase in the size of postsynaptic densities and, by extension, the increase in the number of synaptic AMPA receptors. Since Cch is known to increase dendritic spine size (De Roo et al. 2008), we measured synaptic strength by recording the ratio of AMPA/NMDA receptors–mediated EPSCs amplitudes in CA1 pyramidal neurons. We observed no change in the AMPA/NMDA ratio after stimulating the slices with Cch in the presence of the Inhibitor I (Ctrl: 3.07 ± 0.45, *n* = 4 slices, Inh I + Cch: 3.84 ± 1.2, *n* = 4, *P* = 0.57, *t*-test; Fig. 7*H*).

## Discussion

In this study, we demonstrate that the function of MMP-9 in a cholinergically induced modulation of hippocampal network is to maintain the fine balance between excitatory and inhibitory synaptic transmission. Without MMP-9 activity, Cch stimulation triggers a robust enhancement of synaptogenesis on CA1 pyramidal cells, boosting the excitatory drive onto CA1 neurons.

To study the long-term consequences of cholinergically induced plasticity on MMP-9 function, we used the organotypic hippocampal cultures, in which we investigated MMP-9 activity and the effects of its manipulations on excitatory and inhibitory synaptic transmission. It was reported that acetylcholine stimulation of 1–3-week-old organotypic cultures resulted in a stable induction of gamma rhythm (Schneider et al. 2015). Indeed, our results show that Cch application induced gamma oscillations in organotypic slices, yet we were unable to detect a direct effect of MMP-9 inhibition on the power, frequency, or amplitude of this rhythmic activity (data not shown). Thus, the potentiation of the excitatory transmission caused by MMP-9 inhibition is not a result of any change in the magnitude of Cch-induced oscillations but increased, abnormal excitatory connectivity within the hippocampal slice. Interestingly, Alaiyed et al. (2019) have recently demonstrated that serotonin/norepinephrine reuptake inhibitor venlafaxine-enhanced gamma power was significantly reduced in mice that were deficient in MMP-9, in acute hippocampal slices, which suggests the indirect role of MMP-9 in the modulation of gamma oscillations.

Cholinergic activation triggers a powerful modulation of both excitatory and inhibitory synaptic transmission, coordinating the firing of different populations of neurons, which is essential for the execution of complex behaviors (Moriguchi et al. 2009; Picciotto et al. 2012; Sun and Kapur 2012). In the hippocampus, activation of mAChRs depolarizes pyramidal neurons and increases their excitability (Dodd et al. 1981; Cole and Nicoll 1983; Park and Spruston 2012). Furthermore, since hippocampal GABAergic interneurons are a very heterogeneous population, the effects of mAChRs activation are far more complex. For instance, Cch increases the frequency and amplitude of spontaneous IPSCs but reduces GABA release (Behrends and ten Bruggencate 1993). Such fine-tuning of excitatory and inhibitory transmission has a major consequence on the network level, where cholinergic activation triggers oscillatory activity of interneurons that, in turn, synchronize the firing of pyramidal cells (Cobb et al. 1995). The application of Cch induces the enlargement of dendritic spines on CA1 pyramidal cells, detected 5 h after the stimulation. These spines return to their basal size by 24 h poststimulation (De Roo et al. 2008). Our data measured at 21 h post-Cch stimulation (with and without the Inhibitor I) show a slight enlargement of PSD-95 puncta size that could suggest the increase in AMPA receptor content. Yet, since neither mEPSCs amplitudes increased after Cch nor AMPA/NMDA ratio changed, in aggregate, these findings indicate that there was no increase in the number of AMPARs at 14–22 h post-Cch + Inh I. It is possible, therefore, that we observed a delay in the decrease of spine size that follows the reduction of AMPARs content to their basal state. Together, these results suggest that cholinergically induced increase in the excitatory drive is initially triggered by a transient enhancement of existing synapses and later maintained via the progressive increase in the number of excitatory connections.

In basal synaptic transmission in the hippocampus, the activity of MMP-9 is very low. However, it increases markedly in response to synaptic plasticity induction, as shown in chemically induced and theta-burst stimulation LTP models, and is necessary for the maintenance of late phase of LTP and structural plasticity of dendritic spines (Nagy et al. 2006; Michaluk et al. 2007; Wang et al. 2008; Szepesi et al. 2013; Wiera et al. 2013; Szepesi et al. 2014; Magnowska et al. 2016). In the present study, we observe that, similar to LTP models, Cch-induced activation of the cholinergic system (presumably via mAChRs, since the effect was abrogated by scopolamine) triggers an increase in MMP-9 enzymatic activity that develops further over the course of hours. Surprising finding of pro-MMP-9 and TIMP-1 proteins marked increase at 16 h post-Cch can be explained by the fact that TIMP-1–bound MMP-9 may indeed represent a large portion of total MMP-9 available at that time. Moreover, it has been noted that gel zymography might not be efficient enough at detecting pro-MMP-9 form, as only a fraction of pro-MMP-9 autoactivates after SDS gel electrophoresis (Woessner 1995; Ren et al. 2017). In situ zymography imaging revealed a punctate pattern of DQ gelatin fluorescence, suggesting a synaptic localization of MMP-9 gelatinolytic activity. Consequently, such enhanced MMP-9 action potentiates the excitatory input to a subpopulation of GABAergic cells in stratum radiatum, as demonstrated by the increased mEPSCs frequency. On the other hand, cholinergically induced elevation of MMP-9 activity increased the frequency of mIPSCs recorded in pyramidal CA1 cells, indicating a potentiation of inhibition of pyramidal neurons as well. Hence, we show that cholinergically induced MMP-9 activity affects both the inhibition and excitation of hippocampal circuits, hours after the Cch stimulation.

The proper function of MMP-9 integrates the cholinergically induced changes in the hippocampal network by fine-tuning the activity of excitatory and inhibitory circuitry. However, when blocked by a specific inhibitor, MMP-9 is unable to maintain the homeostasis. This leads to a marked potentiation of excitatory input to pyramidal neurons, observed as an increase in mEPSCs frequency and the number of excitatory synapses on CA1 neurons. Similarly, when using organotypic slices obtained from the MMP-9 KO mice, we observe equally strong, cholinergically induced increase in miniature EPSCs frequency, comparable to the one evoked by the inhibition of MMP-9 activity. Previous studies have localized the activity-dependent release of MMP-9 to the dendritic spines of excitatory neurons (Wilczynski et al. 2008; Szepesi et al. 2013; Stawarski et al. 2014; Szepesi et al. 2014). Moreover, MMP-9 activity was shown to be essential for seizure-induced synaptic pruning in rodent models of epileptogenesis (Wilczynski et al. 2008). In this study, we observed that cholinergic stimulation induces MMP-9 activity and potentiates the excitatory synaptogenesis on CA1 neurons. We hypothesize that the role of MMP-9 in this process is in fact to allow the removal of the excess of excitatory connections. The inhibitor I blocks the MMP-9–mediated pruning of excitatory synapses, which leads to an abnormal buildup of excitatory connections on pyramidal neurons, as revealed by immunostaining and the analysis of the density of dendritic spines. Thus, our data indicate that without MMP-9 activity, the cholinergically induced rhythmic activity within the hippocampal circuitry leads to an enhanced synaptogenesis and potentiated excitatory drive.

## Concluding Remarks

**Figure 8:**
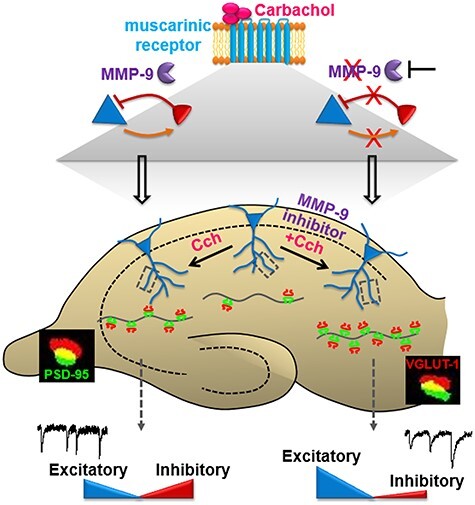
Graphical summary: cholinergically induced MMP-9 activity mediates the modulation of the excitatory and inhibitory synaptic transmission. Activation of cholinergic muscarinic receptors triggers an enhancement of inhibitory currents input onto hippocampal CA1 pyramidal neurons (blue), and in turn, a potentiation of fast-spiking inhibitory neurons (red) via the increase of excitatory currents input. Both synaptic connections from inhibitory to excitatory neurons and vice versa are facilitated by the activity of MMP-9. Blocking the MMP-9 activity leads to an even larger boost of excitatory currents input onto the CA1 pyramidal neurons, while preventing the inhibitory currents potentiation. These unbalanced excitatory and inhibitory transmissions strongly potentiate the growth of synaptic connections in the CA1 pyramidal neurons, while only a moderate increase in synapse number is induced by cholinergic activation when MMP-9 is active.

This research reveals a novel function of MMP-9 in mediating the balance between excitatory and inhibitory synaptic transmission, following cholinergically induced plasticity in the hippocampus. Cch treatment, which induces gamma oscillations in organotypic hippocampal cultures, increases MMP-9 activity and potentiates both inhibitory and excitatory inputs to CA1 pyramidal neurons. When, however, the MMP-9 function is blocked, the Cch-induced plasticity drives hippocampal connectivity toward increased excitation, via abnormal enhancement of synaptogenesis on the CA1 principal neurons (Fig. 8). Future studies shall reveal the molecular mechanisms of MMP-9 function in orchestrating the activity of inhibitory circuitry and pinpoint the exact targets and temporal precision of MMP-9 proteolytic action during cholinergic modulation of hippocampal circuitry.

## Author Contributions

A.S., L.K., and A.B. designed the experiments. A.S., D.L., K.N., and B.B. performed all experiments on hippocampal organotypic slice cultures. A.S., D.L., K.N., B.B., P.K.G., B.C., and T.K. analyzed the data. A.B. and L.K. supervised the studies. All authors contributed to data interpretation and manuscript review. A.S. and A.B. wrote the manuscript with input from all authors.

## Funding

This work was supported by the National Science Centre (NCN) Poland (MAESTRO 2017/26/A/NZ3/00379 to L.K.) and the Foundation for Polish Science (MAB/2018/10 to A.B.); ``Nencki-EMBL Center of Excellence for Neural Plasticity and Brain Disorders: BRAINCITY'' project is carried out within the International Research Agendas programme of the Foundation for Polish Science co-financed by the European Union under the European Regional Development Fund.

## Notes

*Conflict of Interest*: The authors declare no competing interests.

## Supplementary Material

Supplementary_Results_bhab050
