# Corrigendum to: Inhibition of Matrix Metalloproteinase 9 Activity Promotes Synaptogenesis in the Hippocampus

**DOI:** 10.1093/cercor/bhab105

**Published:** 2021-04-09

**Authors:** Ahmad Salamian, Diana Legutko, Klaudia Nowicka, Bogna Badyra, Paulina Kaźmierska-Grębowska, Bartosz Caban, Tomasz Kowalczyk, Leszek Kaczmarek, Anna Beroun

**Affiliations:** 1 Laboratory of Neurobiology, Nencki-EMBL Center of Excellence for Neural Plasticity and Brain Disorders: BRAINCITY, Nencki Institute of Experimental Biology of the Polish Academy of Sciences, Warsaw, Poland; 2 Laboratory of Molecular Basis of Behavior, Nencki Institute of Experimental Biology of the Polish Academy of Sciences, Warsaw, Poland; 3 Department of Neurobiology, Faculty of Biology and Environmental Protection, University of Lodz, Lodz, Poland; 4 Laboratory of Neuronal Plasticity, Nencki-EMBL Center of Excellence for Neural Plasticity and Brain Disorders: BRAINCITY, Nencki Institute of Experimental Biology of the Polish Academy of Sciences, Warsaw, Poland

In the originally published version of this manuscript, there was an error in Figure 2. In panel D of the figure, one of the asterisks that shows significance was placed in the wrong spot. Figure 2 has been updated as follows online:

**Previous version**

**Figure 2:**
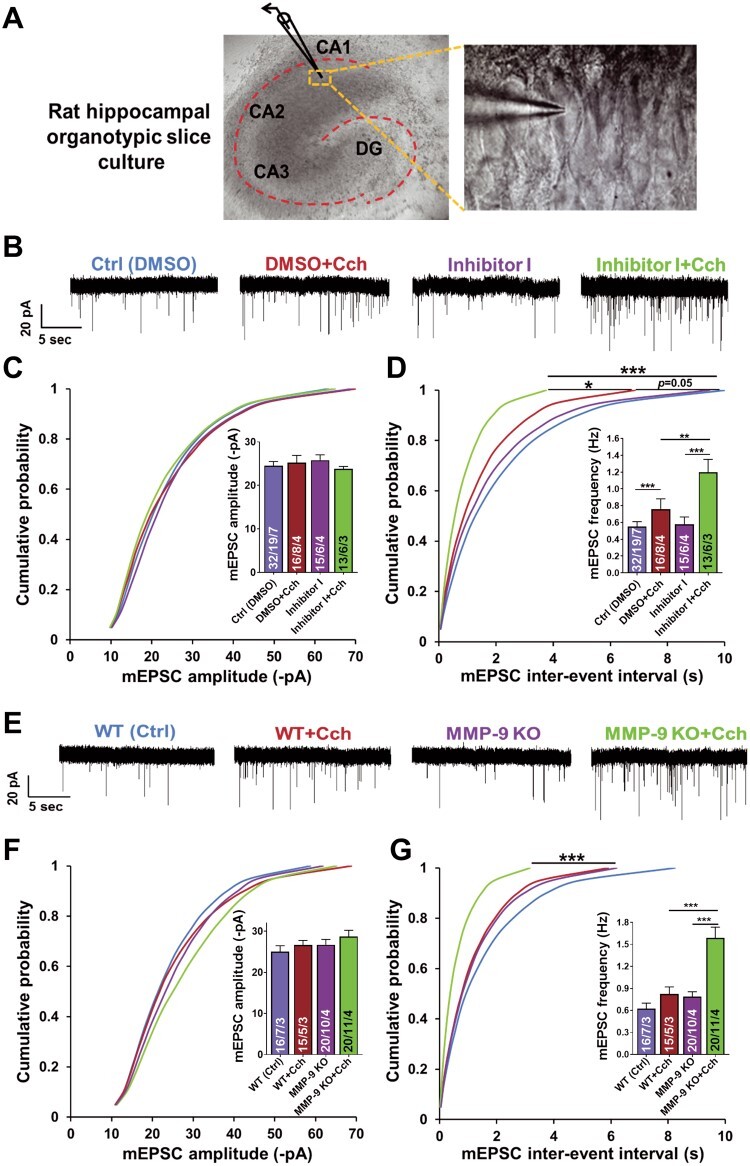
Chemical inhibition or genetic ablation of MMP-9 results in a dramatic increase of the frequency of AMPAR mEPSCs induced by Cch at hippocampal CA1 excitatory synapses. (A) A DIC image of rat hippocampal organotypic slice culture displaying a patched pyramidal cell in the CA1 region. (B) Representative traces of mEPSCs from different experimental groups. (C) Cumulative distributions of event amplitudes and mean event amplitudes (inset) display no effect of Cch (10 μM) on mEPSCs amplitude. (D) Cumulative distributions of interevent intervals and mean event frequencies (inset) show an increase of mEPSCs frequency after Cch and further increase by inhibition of MMP-9 activity. (E) Representative traces of mEPSCs. (F) Cumulative distributions of event amplitudes and mean event amplitudes (inset) show no difference of AMPAR mEPSCs amplitude between WT or MMP-9 KO slices, control and Cch treated (mouse hippocampal organotypic slice cultures). (G) Cumulative distributions of interevent intervals and mean event frequencies (inset) of MMP-9 KO slices reveal a dramatic increase of AMPAR mEPSCs frequency after Cch stimulation compared to KO and WT controls. Statistical comparisons on cumulative distributions were performed with Kolmogorov-Smirnov test and nested one-way ANOVA followed by Sidak's test for bar graph inserts (mEPSCs frequency: F3,34 = 12.70, Inh I+Cch vs. Ctrl P<0.001, Inh I+Cch vs. Cch P = 0.007, Inh I+Cch vs. Inh I P = 0.0002; mEPSCs frequency in MMP-9 KO: F3,29 = 17.04,MMP-9 KO+Cch vs. WT+Cch P = 0.0003,MMP-9 KO+Cch vs.MMP-9 KO P<0.0001). Numbers inside each bar represent the numbers of neurons/slices/cultures. The number of slices was used for statistical analysis (P value: ^*^P<0.05, ^*^^*^P<0.01, ^*^^*^^*^P<0.001).

**Corrected version**

**Figure 2:**
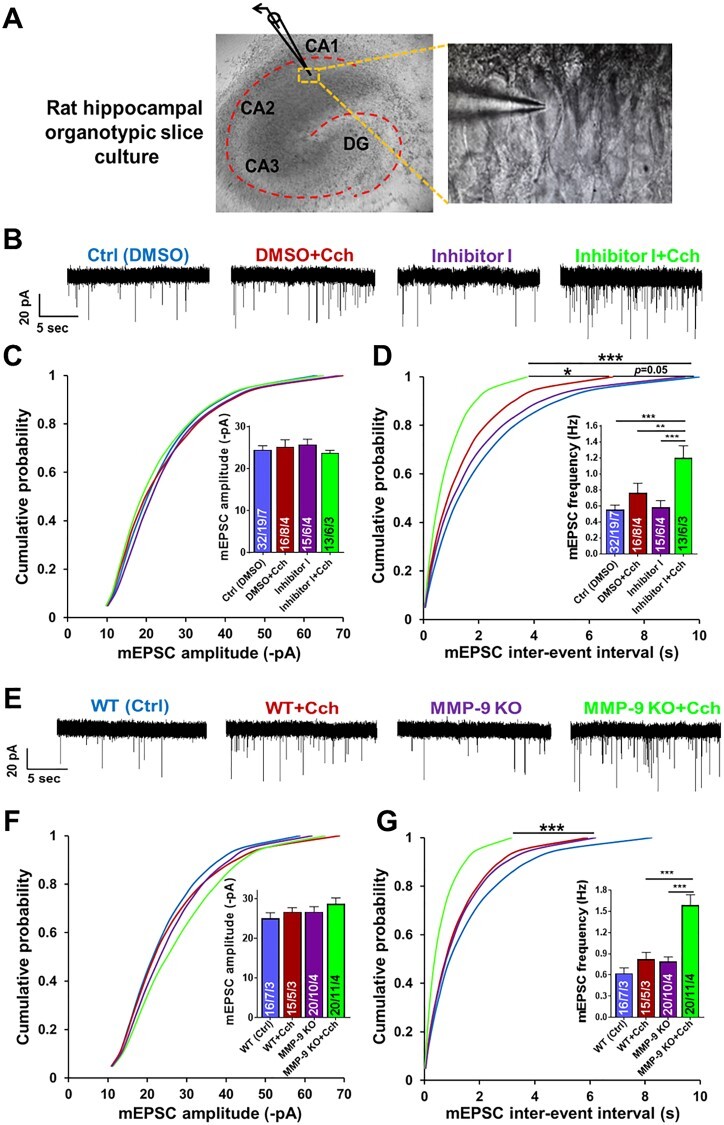
Chemical inhibition or genetic ablation of MMP-9 results in a dramatic increase of the frequency of AMPAR mEPSCs induced by Cch at hippocampal CA1 excitatory synapses. (A) A DIC image of rat hippocampal organotypic slice culture displaying a patched pyramidal cell in the CA1 region. (B) Representative traces of mEPSCs from different experimental groups. (C) Cumulative distributions of event amplitudes and mean event amplitudes (inset) display no effect of Cch (10 μM) on mEPSCs amplitude. (D) Cumulative distributions of interevent intervals and mean event frequencies (inset) show an increase of mEPSCs frequency after Cch and further increase by inhibition of MMP-9 activity. (E) Representative traces of mEPSCs. (F) Cumulative distributions of event amplitudes and mean event amplitudes (inset) show no difference of AMPAR mEPSCs amplitude between WT or MMP-9 KO slices, control and Cch treated (mouse hippocampal organotypic slice cultures). (G) Cumulative distributions of interevent intervals and mean event frequencies (inset) of MMP-9 KO slices reveal a dramatic increase of AMPAR mEPSCs frequency after Cch stimulation compared to KO and WT controls. Statistical comparisons on cumulative distributions were performed with Kolmogorov-Smirnov test and nested one-way ANOVA followed by Sidak's test for bar graph inserts (mEPSCs frequency: F3,34 = 12.70, Inh I+Cch vs. Ctrl P<0.001, Inh I+Cch vs. Cch P = 0.007, Inh I+Cch vs. Inh I P = 0.0002; mEPSCs frequency in MMP-9 KO: F3,29 = 17.04,MMP-9 KO+Cch vs. WT+Cch P = 0.0003,MMP-9 KO+Cch vs.MMP-9 KO P<0.0001). Numbers inside each bar represent the numbers of neurons/slices/cultures. The number of slices was used for statistical analysis (P value: ^*^P<0.05, ^*^^*^P<0.01, ^*^^*^^*^P<0.001).

