# Supplementary material for: Inhibition of Matrix Metalloproteinase 9 Activity Promotes Synaptogenesis in the Hippocampus
Source: Cereb Cortex. 2021 Mar 19;31(8):3804–19. doi: 10.1093/cercor/bhab050 (PMC8258443; doi:10.1093/cercor/bhab050)
Supplement: Supplementary_Results_bhab050 [file supplementary_results_bhab050.docx]

**Supplementary Results**


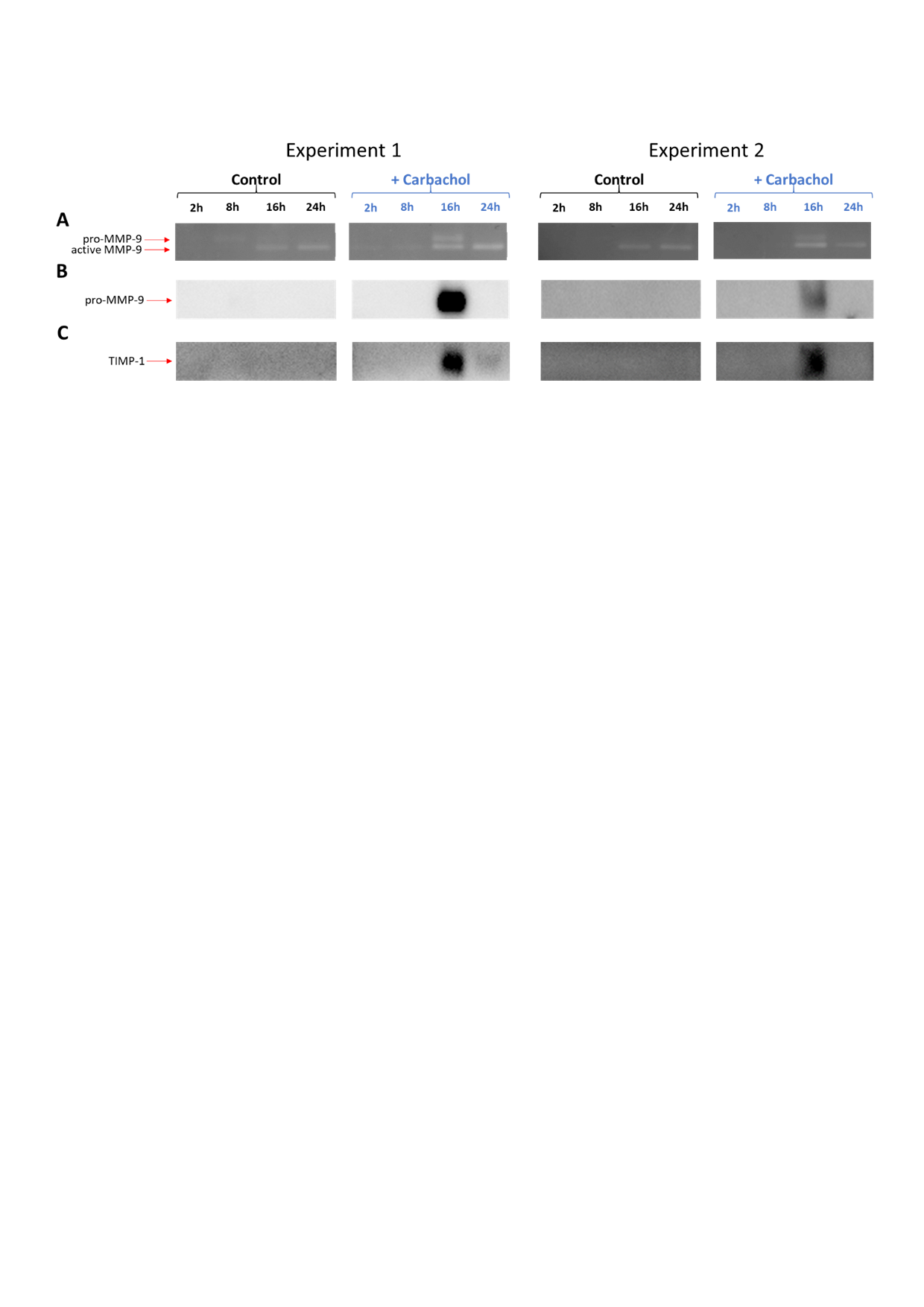


**Fig. S1. Comparative assessment of the MMP-9 enzymatic activity and protein, along with TIMP-1 protein levels.**

(A) Gelatin zymography from two independent experiments, demonstrating the enzymatic activity of MMP‑9 in equal amounts of conditioned ACSF from different time points up to 24 h after Cch stimulation. Two forms of MMP-9, pro- and active enzyme, were detected according to the molecular weight; please note the reproducibly marked increase in proMMP-9 at 16 h post-Cch. (B) Western Blot analysis of MMP-9 protein levels. Comparing to gelatin zymography, and considering the molecular weight, selected antibody bound specifically to pro-MMP-9 form of enzyme with a marked peak at 16 h after Cch treatment. (C) Western Blot analysis of TIMP-1 demonstrating that the peak of the protein expression coincided with the highest levels of pro-MMP9.
